# Fetal gestational age prediction via shape descriptors of cortical development

**DOI:** 10.3389/fped.2024.1471080

**Published:** 2024-11-20

**Authors:** Tommaso Ciceri, Letizia Squarcina, Alessandra Bertoldo, Paolo Brambilla, Simone Melzi, Denis Peruzzo

**Affiliations:** ^1^ NeuroImaging Lab, Scientific Institute IRCCS Eugenio Medea, Bosisio Parini, Italy; ^2^ Department of Information Engineering, University of Padua, Padua, Italy; ^3^ Department of Pathophysiology and Transplantation, University of Milan, Milan, Italy; ^4^ Neuroscience Center, University of Padua, Padua, Italy; ^5^ Department of Neurosciences and Mental Health, Fondazione IRCCS Ca’ Granda Ospedale Maggiore Policlinico, Milan, Italy; ^6^ Department of Informatics, Systems and Communication (DISCo), University of Milano-Bicocca, Milan, Italy

**Keywords:** fetal brain, gestational age prediction, shape descriptors, cortical surface, MRI

## Abstract

**Introduction:**

Gyrification is the intricate process through which the mammalian cerebral cortex develops its characteristic pattern of sulci and gyri. Monitoring gyrification provides valuable insights into brain development and identifies potential abnormalities at an early stage. This study analyzes the cortical structure in neurotypical and pathological (spina bifida) fetuses using various shape descriptors to shed light on the gyrification process during pregnancy.

**Methods:**

We compare morphometric properties encoded by commonly used scalar point-wise curvature-based signatures—such as mean curvature (H), Gaussian curvature (K), shape index (SI), and curvedness (C)—with multidimensional point-wise shape signatures, including spectral geometry processing methods like the Heat Kernel Signature (HKS) and Wave Kernel Signature (WKS), as well as the Signature of Histograms of Orientations (SHOT), which combines histogram and signature techniques. These latter signatures originate from computer graphics techniques and are rarely applied in the medical field. We propose a novel technique to derive a global descriptor from a given point-wise signature, obtaining GHKS, GWKS, and GSHOT. The extracted signatures are then evaluated using Support Vector Regression (SVR)-based algorithms to predict fetal gestational age (GA).

**Results:**

GSHOT better encodes the GA to other global multidimensional point-wise shape signatures (GHKS, GWKS) and commonly used scalar point-wise curvature-based signatures (C, H, K, SI, FI), achieving a prediction *R*^2^ of 0.89 and a mean absolute error of 6 days in neurotypical fetuses, and a *R*^2^ of 0.64 and a mean absolute error of 10 days in pathological fetuses.

**Conclusion:**

GSHOT provides researchers with an advanced tool to capture more nuanced aspects of fetal brain development and, specifically, of the gyrification process.

## 1 Introduction

Magnetic resonance imaging (MRI) has become a pivotal tool for the study of brain morphology and understanding structural alterations associated with various pathological conditions. Various geometric quantities can be exploited to summarize the morphometric information, providing valuable insights into population-based studies, contributing to our understanding of brain-related disorders, and paving the way for more personalized approaches to diagnosis and treatment. Advanced shape analysis techniques allow us to explore new dimensions of brain morphology beyond traditional measures (e.g., brain volumes and surface areas). Some of these techniques utilize spectral (1) and local extrinsic geometric (2) properties to gain deeper insights into brain shape characteristics. Spectral shape analysis techniques involve analyzing the shape of an object based on its spectral properties, and this is typically accomplished by encoding the shape through a differential operator and computing its eigendecomposition. On the other hand, local geometric properties focus on analyzing the shape at a more localized and detailed level, typically involving specific quantities such as curvature, surface normals, or deformations at specific points or regions of the shape. In this fashion, shapes can be compared by measuring similarities between these features. Thus, the efficacy of shape descriptors can be assessed in terms of discriminativeness and robustness against shape variations due to noise or deformations (3).

In this work, we analyze the fetal brain cortical structure using different shape descriptors to enhance our comprehension of the fetal brain gyrification process, i.e., the formation of gyri and sulci. The gyrification process plays a crucial role in brain development, contributing significantly to overall growth, organization, and functionality. Just like fetal ultrasound provides an estimate of gestational age (GA) by measuring basic morphometric features (such as skull size or femur length), tools linking brain morphological MR images to the central nervous system development can be a valuable resource for monitoring pregnancy and detecting fetal diseases in their initial stages. Here, we estimate the fetus GA in weeks, comparing the cortical structure morphometric properties encoded with the commonly used scalar point-wise curvature-based descriptors to those derived via multidimensional point-wise shape signatures which are widely used in computer graphics analysis. We examine several scalar point-wise signatures based on curvature: mean curvature (H), which measures extrinsic curvature or folding; Gaussian curvature (K), which measures intrinsic curvature or distortion; shape index (SI) and folding index (FI), indicators of shape and folding patterns (4). Moreover, we consider the curvedness (C), a signature incorporating information from both H and K, a valuable measure of the gyrification process (5, 6). On the other hand, we examine three different multidimensional point-wise shape signatures that are rarely applied in the medical field: the Heat Kernel Signature [HKS, (7)], the Wave Kernel Signature [WKS, (8)], and the Signature of Histograms of OrienTations [SHOT, (2)]. HKS is derived from the heat equation, a partial differential equation that describes the heat diffusion across the surface over time. Similarly, WKS is derived from the solution of another partial differential equation, the wave equation, which describes the evolution of waves across the surface over time. SHOT is computed by dividing the neighborhood around each point into multiple cells and calculating histograms of relative orientations of the normals in each cell. These histograms are then concatenated to obtain the final signature, which results in a compact representation of the local geometric properties of the shape. In the last years, several studies have been proposed to analyze fetal brain gyrification by extracting cortical surface morphometric properties. In this context, scalar point-wise curvature-based measures are the gold standard for assessment of neurodevelopment (5, 6, 9–14). Other novel techniques based on sulcal pattern analysis can be employed to observe geometric and topological patterning of early sulcal folds, including 3D positions, sulcal basin surface area, and depth (4, 15–17).

Furthermore, we define a novel procedure to extract a global encoding framework from these multidimensional point-wise shape signatures, leading to their global version. We namely refer to the global descriptors produced through our pipeline as Global plus the name of the input pointwise descriptor we use, Global Heat Kernel Signature (GHKS), Global Wave Kernel Signature (GWKS), and Global Signature of Histograms of OrienTations (GSHOT). The global descriptor has several excellent advantages such as it allows for shape comparisons using minimal shape preprocessing, it is robust to noise since it implicitly employs surface smoothing by neglecting higher frequencies of the shape, and finally, it encodes isometric invariance properties of the shape, which are crucial to deal with shape deformations.

We tested our *descriptors* in the context of the fetal brain gyrification process. A linear Support Vector Regression (SVR)-based approach (18) was employed to predict the fetus GA from the cortical structure morphometric properties encoded by descriptors. Experiments on a public dataset of 80 fetuses (*n* = 31 neurotypical and *n* = 49 pathological (19), and two public atlases of 18 and 16 fetuses (20, 21) showed promising prediction results in distinguishing the fetal brain gyrification process.

## 2 Methods

The proposed approach comprises five main steps: data gathering, cortical structure reconstruction, computation of shape descriptors, and GA prediction.

### 2.1 Data

We included data from different sources in this study. In particular, we used two publicly available fetal brain atlases (20, 21) and one publicly available fetal brain dataset (19). For each source, we used all the provided data without assessing the quality of the fetal brain high-resolution reconstruction and tissue segmentation. A brain atlas is a digital representation of the human brain population, which highlights common structural features and provides a reference point for researchers and clinicians to compare and analyze specific brain regions. On the other hand, a brain dataset refers to a collection of brain images of real fetuses, thus characterized by unique variations.

The fetal brain atlas introduced by Gholipour et al. (20) (hereafter, “CRL atlas”) is defined at the GA range of 21–38 weeks. It consists of an age-specific T2-weighted (T2w) template and label images of 124 brain tissues, including gray matter (GM) and white matter (WM). The fetal brain atlas introduced by Uus et al. (21) (hereafter, “dHCP fetal atlas”) is defined at 21–36 weeks. It includes age-specific T2w templates and 19 brain tissue labels, separate for each hemisphere.

On the other hand, the fetal brain dataset introduced initially by Payette et al. (22) and later updated (19) (hereafter, “FeTA dataset”) consists of MRI-reconstructed images of 80 fetuses (*n* = 49 pathological and *n* = 31 neurotypical) defined in the GA range of 20–35 weeks. Each subject was released with a T2w template brain reconstruction (reconstructed with either NiftyMIC^1^, MIALSRTK^2^, or Simple IRTK^3^) with the corresponding seven brain tissue label images. Pathological subjects included fetuses with spina bifida either before or after fetal spinal lesion repair surgery, as these were the only publicly available pathological datasets (23). A summary of the available cohort of fetuses is reported in Table 1.

**Table 1:** Summary of the publicly available fetal brain MRI atlases and dataset used in our study.

Data	MRI contrast	Tissue labels	GA range	Cohort	Public link
CRL atlas	T2w	124	21–38 weeks	18	http://crl.med.harvard.edu/research/fetal_brain_atlas
dHCP fetal atlas	T2w	19	21–36 weeks	16	https://gin.g-node.org/kcl_cdb/fetal_brain_mri_atlas
FeTA dataset	T2w	7	20–35 weeks	80 (31 neurotypical 49 pathological)	https://www.synapse.org/#!Synapse:syn25649159/wiki/610007

### 2.2 Cortical structure reconstruction

**Figure 1:**
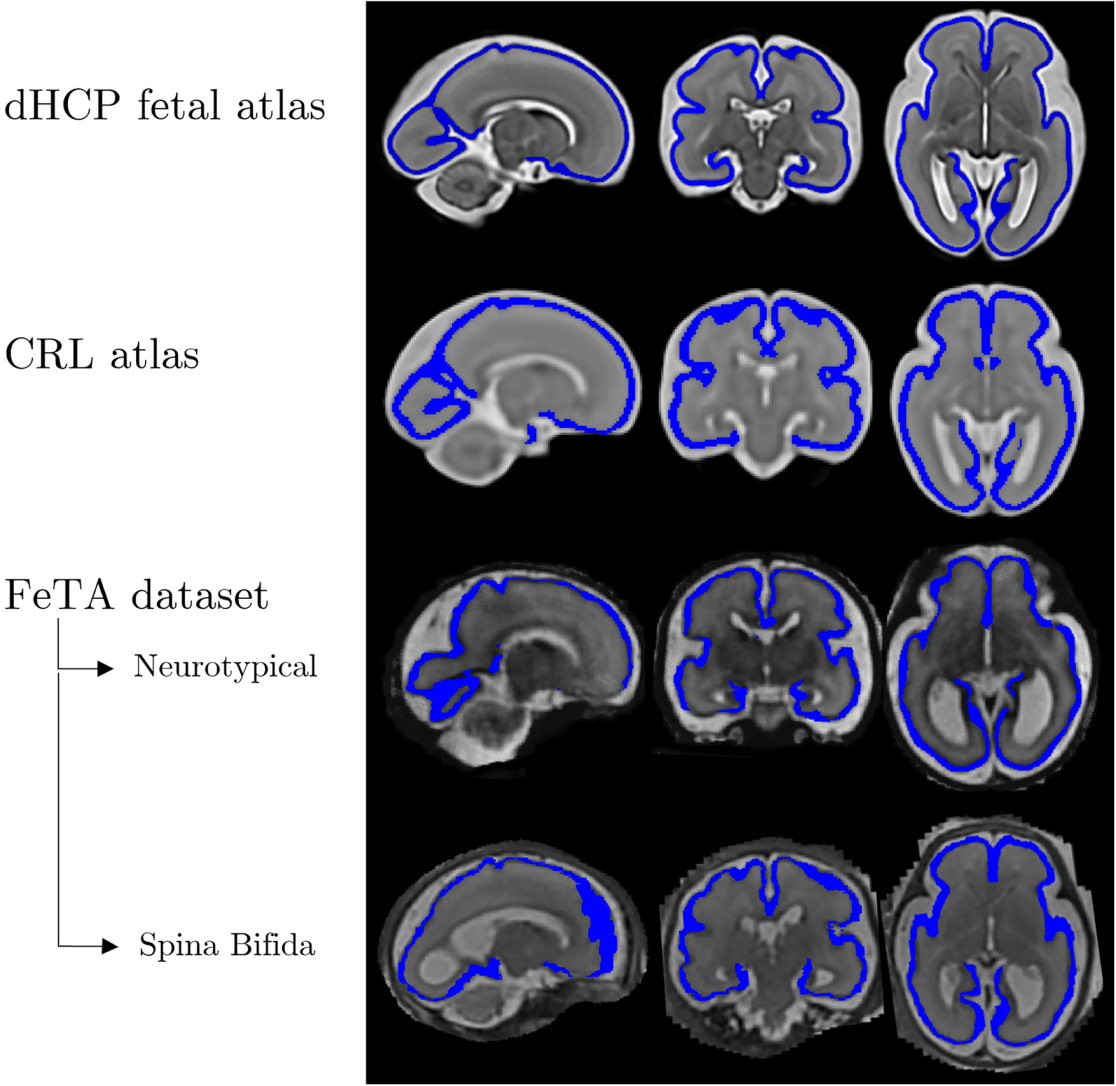
Examples of brain images and corresponding cortical plate segmentations in various atlases (20, 21) and datasets (19) for a 28-week gestational age fetus. Structural anatomical (grayscale) and cortical plate segmentation (blue) overlaid. From left to right, the fetal brains are displayed in three different orientations - sagittal, coronal, and axial.

Our study focused on the cortical structure of the fetal brain, which is defined as the external layer of the parenchymal tissue and will become the cortical GM in the mature brain (Figure 1). During embryonic development, the brain is surrounded by a thin layer (darker than other tissues in the T2w images) called the cortical plate (CP). In the beginning, the CP is a flat and smoothed structure; as the brain grows and enlarges, it thickens and differentiates into different cortical layers. Visually, this results in folds, or gyri, and grooves, or sulci, that give the brain its characteristic wrinkled appearance. By the end of fetal development, the differentiated CP becomes the outermost layer of the brain, known as the cortical GM.

The CP folding process can be monitored using its boundary surfaces, i.e., the external and internal surfaces. The external surface separates the parenchyma from the cerebrospinal fluid. On the other hand, the inner surface divides the CP and the WM structure. We decided to focus on the inner cortical surface since the interface between WM and cortex is more stable and less prone to segmentation errors due to partial volume effects than the cortex-cerebrospinal fluid interface (15).

**Figure 2:**
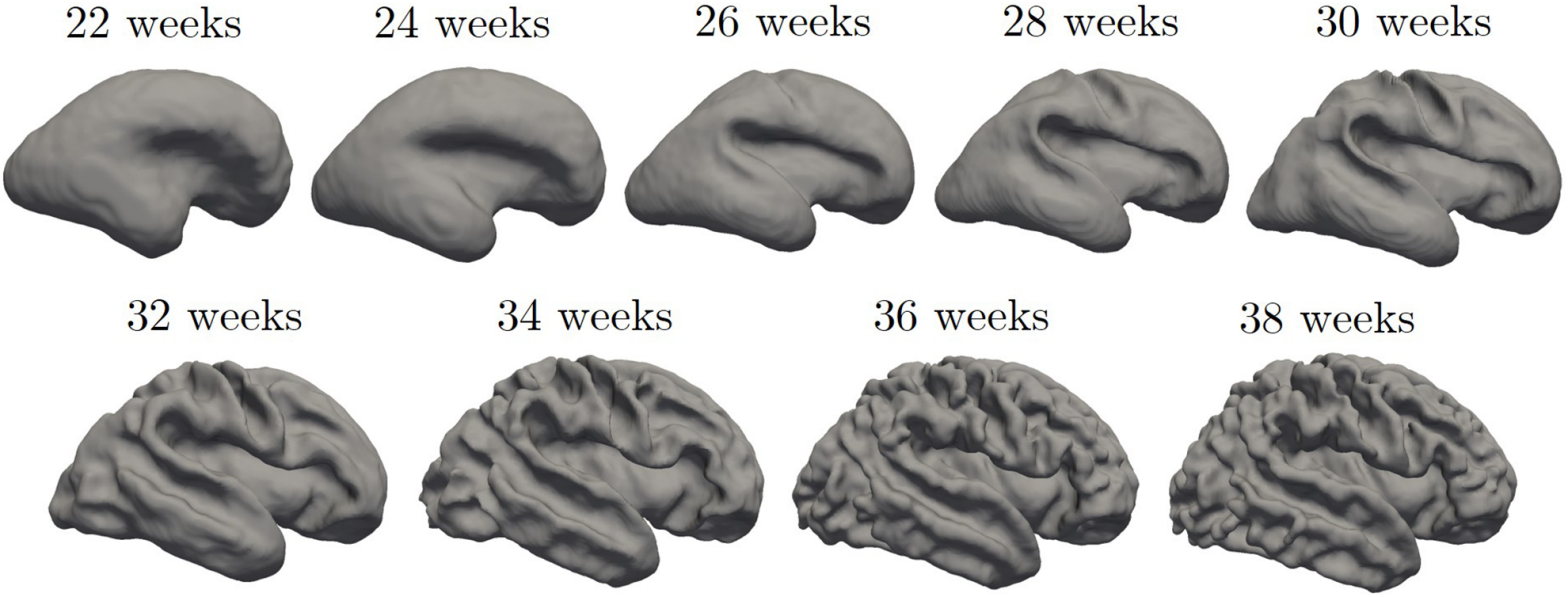
Inner cortical surface of the fetal brain from 28 to 38 gestational weeks. The surfaces depicted in the figure are generated from the previously quoted Gholipour et al. (20) atlas.

For each data set, we generated the inner cortical volume by merging the already validated tissue segmentation labels, which encompass the WM to the inner structures of the brain. Although the different datasets were generated using different segmentation protocols (19–21), they all include the inner cortical surface as boundary between structures, enabling consistent identification in each dataset used. Consequently, the volume obtained for each fetus was binarized and underwent manual refinement to remove any erroneous segmented components (i.e., voxel connected to the main WM mask by a single vertex). Subsequently, we extracted (MATLAB *isosurface* function) a triangular mesh representing the boundary of the binary image, i.e., the inner surface of the CP. Furthermore, we employed Freesurfer (surfer.nmr.mgh.harvard.edu) to geometrically smooth the resulting mesh, removing noise and minor geometric alterations. Figure 2 shows an example of the fetal brain's inner cortical surfaces across different gestational weeks.

### 2.3 Shape descriptors

To capture the most informative intrinsic geometric properties of the inner cortical surface shape, we computed both scalar point-wise curvature-based signatures (C, H, K, SI, FI) and multidimensional point-wise shape signatures (HKS, WKS, SHOT).

Scalar point-wise curvature-based signatures are computed for each vertex of the surface mesh using the FreeSurfer function *mris_curvature_stats* (10). Subsequently, we derived a global description of the derived signature by computing the frequency over a 100 bins discretization (MATLAB *histcounts* function), and we normalized the derived distribution for the number of associated vertices, which is an intrinsic characteristic of each fetus (24).

**Figure 3:**
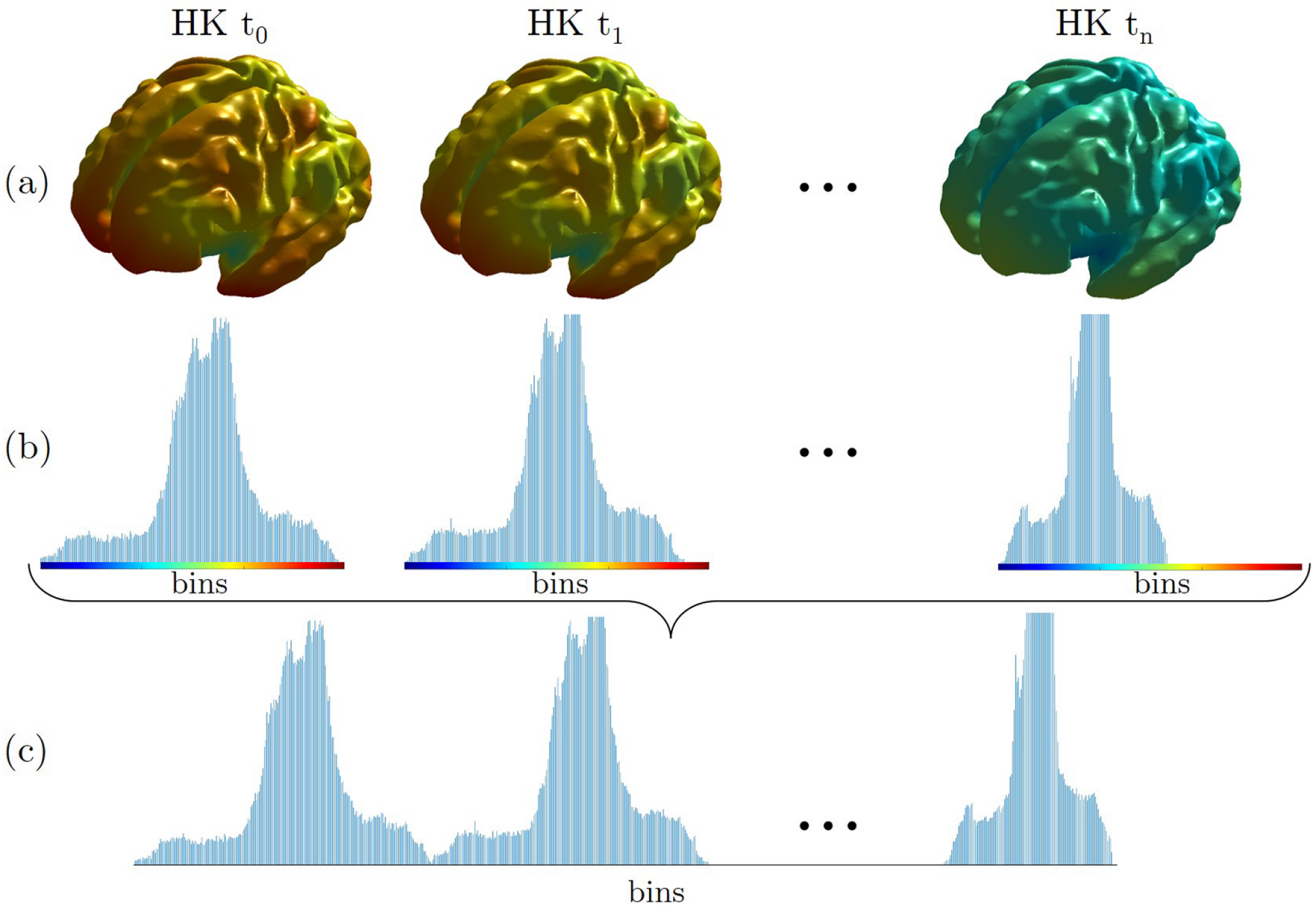
An example of a global descriptor construction for the HKS signature (GHKS). **(a)** Each point of the inner cortical shape of the brain is colored according to the heat kernel (HK) value at time *t_i_*. **(b)** These values are then gathered into histograms for each scale *t_i_*. **(c)** The histograms are concatenated, leading to the global signature. The brain's surfaces shown in the figure are generated from a 33-week fetus of the previously quoted Gholipour et al. (20) atlas.

Multidimensional point-wise shape signatures are computed accordingly. In detail, the HKS and WKS descriptors are implemented with an in-house MATLAB code. Here, we used *k* = 100 eigenvalues and scaled the temporal domain logarithmically in *n* = 10 time values, as suggested by Sun et al. (7). On the other hand, the SHOT descriptor is implemented in Pyshot, a Python library publicly available on GitHub (https://github.com/uhlmanngroup/pyshot). As for the point-wise curvature-based signatures, we derived a global description from HKS, WKS, and SHOT features, computing their distribution on a 100 bins discretization, and we normalized the derived distribution for the number of associated vertices. Finally, we concatenated the obtained distributions for each time point to derive its global signature similar to what was proposed in (3). A schematic example of the proposed global HKS is presented in Figure 3. This schema is adopted for each multidimensional signature investigated to obtain its global version.

To aid in the data visualization of the signatures extracted by each descriptor, we performed a Principal Component Analysis (PCA). We derived a 2D scatter plot representing each descriptor's first and second principal components. These components contain the most relevant variations shown in the dataset, proving its capability to encode the changes in shape.

### 2.4 Gestational age prediction

We implemented a ML experiment to investigate whether and which derived signatures include the information associated with CP development, and if they can be used to predict a subject's GA.

We used a SVR algorithm to predict the GA in weeks for each image sample from the features extracted with the shape descriptor from the inner cortical surface mesh. SVR is one of the most powerful supervised machine learning approaches used for regression tasks (25), which aims to find a hyperplane maximizing the margin while minimizing errors in a high-dimensional feature space. It is an extension of the support vector machine classification algorithm but it predicts continuous output values instead of class labels. In the present study, only linear kernels were employed since nonlinear methods may require sample sizes that are too large to generalize well (26). Furthermore, we decided to use the z-score method to normalize the data, estimating the mean and the standard deviation on the training set and applying them to normalize the test set. We trained each SVR model on the signatures extracted from the atlases (20, 21) and tested it on the signatures extracted from the FeTA dataset (19). We evaluated the goodness-of-fit of the SVR models by measuring the mean absolute error (MAE), the root mean square error (RMSE), which is more sensitive to outliers than MAE, and the coefficient of determination (*R*^2^), which denotes the amount of variation in potential new observations. The concordance between predicted and true GA was determined using Lin's concordance correlation coefficient, with strength of agreement assessed by McBride's criteria as follows: poor, <0.90; moderate, 0.90–0.95; substantial, 0.95–0.99; almost perfect >0.99 (27, 28).

## 3 Results

The proposed GA prediction method was employed for the characterization of the fetal brain gyrification process occuring during pregnancy. A dataset of 114 images (*n* = 31 neurotypical and *n* = 49 pathological from public dataset, and *n* = 34 neurotypical from online available atlases) has been evaluated. After the surface construction, the scalar point-wise curvature-based signatures (C, H, K, SI, FI) and the global multidimensional point-wise shape signatures (GHKS, GWKS, and GSHOT) were computed and normalized by the z-score technique. The GA prediction procedure is employed as described in Section 2.4. Notably, each linear-based kernel SVR algorithm was trained on the shape signatures extracted from the inner cortical surface of fetuses included in atlases (20, 21) and tested on the fetuses included in the public dataset (19), differentiating between neurotypical and pathological fetuses. Table 2 shows the performance of the individual SVR models in predicting GA for neurotypical fetuses. GSHOT outperforms other shape descriptors, both the scalar point-wise curvature-based signatures and the global multidimensional point-wise shape signatures, achieving a prediction *R*^2^ of 0.89 and a corresponding MAE of 6.3 days. FI is the best scalar point-wise curvature-based descriptor, achieving prediction performance comparable to GSHOT. Notably, it achieved a prediction *R*^2^ of 0.75 and a MAE of 9.9 days. According to Lin's concordance correlation coefficient, the GSHOT model demonstrated *substantial* agreement between descriptor-based GA predictions and GA ground truths (ρc = 0.95, 95% CI = 0.89–0.97), whereas the FI model showed *poor* agreement (ρc = 0.84, 95% CI = 0.72–0.91).

**Table 2:** Gestational age prediction in neurotypical fetuses, expressed in weeks.

Performance metric [weeks]	Scalar point-wise curvature-based signatures					Global multidimensional point-wise shape signatures		
	C	H	K	SI	FI	GHKS	GWKS	GSHOT
RMSE	2.81	4.81	4.11	3.87	1.76	2.04	2.36	**1**.**18**
MAE	2.40	4.32	3.52	3.53	1.41	1.63	1.65	**0**.**91**
*R* ^2^	0.36	−0.86	−0.36	−0.20	0.75	0.67	0.55	**0**.**89**

The goodness-of-fit of the individual linear-SVR models is evaluated by measuring the mean absolute error (MAE), the root mean square error (RMSE), and the coefficient of determination (*R*^2^). The scalar point-wise curvature-based signatures (C, H, K, SI, FI) and the global multidimensional point-wise shape signatures (GHKS, GWKS, GSHOT) are compared.

The best-performing metrics are shown in bold.

Table 3 shows the GA prediction performance of the considered individual SVR models tested on the pathological subset of the FeTA dataset. The results highlight a larger prediction error compared to the neurotypical subset of the FeTA dataset. Similarly, GSHOT outperforms other descriptors, achieving a prediction R^2^ of 0.64 and a corresponding MAE of 10.1 days. However, the agreement between predictions and ground truths for this model was *poor* based on Lin's concordance correlation coefficient (ρc = 0.80, ^95%^CI = 0.70–0.87).

**Table 3:** Gestational age prediction in pathological (spina bifida) fetuses, expressed in weeks.

Performance metric [weeks]	Scalar point-wise curvature-based signatures					Global multidimensional point-wise shape signatures		
	C	H	K	SI	FI	GHKS	GWKS	GSHOT
RMSE	2.96	8.50	6.25	6.04	3.27	3.31	3.53	**1**.**87**
MAE	2.62	8.10	5.75	5.55	2.47	2.84	2.71	**1**.**44**
*R* ^2^	0.10	−6.46	−3.03	−2.77	−0.10	−0.13	−0.28	**0**.**64**

The goodness-of-fit of the individual linear-SVR models is evaluated by measuring the mean absolute error (MAE), the root mean square error (RMSE), and the coefficient of determination (*R*^2^). The scalar point-wise curvature-based signatures (C, H, K, SI, FI) and the global multidimensional point-wise shape signatures (GHKS, GWKS, GSHOT) are compared.

The best-performing metrics are shown in bold.

**Figure 4:**
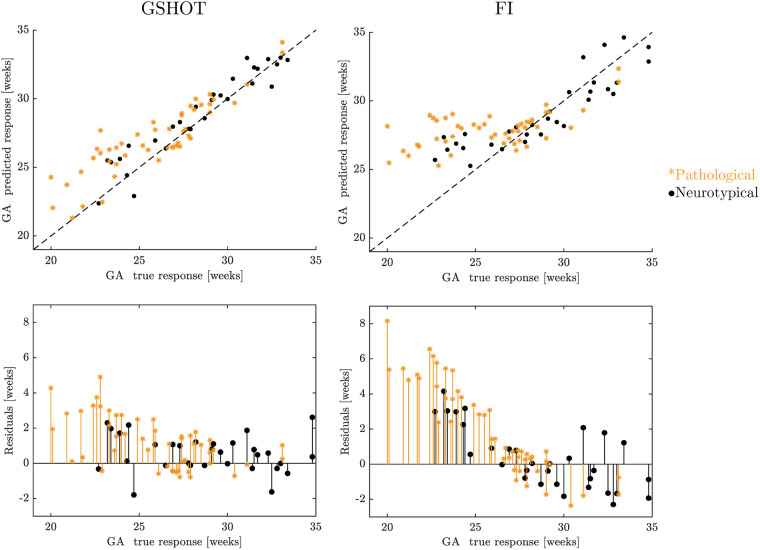
A visualization of the results in GA prediction using GSHOT and FI. The figure displays the true vs. predicted response plots at the top and the residual plots at the bottom. Black points represent neurotypical fetuses, while pathological (spina bifida) fetuses are described by blue stars. The FeTA dataset was used for GA prediction (19).

In Figure 4, the GA prediction obtained with the best global multidimensional point-wise shape signature (GSHOT) and with the best scalar point-wise curvature-based signature (FI) is visualized in the true vs. predicted response plot (top row), and the prediction model is evaluated using the residual plot (bottom row). All the GSHOT points are close to the diagonal line, suggesting an excellent estimation of the SVR model. Moreover, the points of FI are more dispersed than those of GSHOT. Finally, the residuals obtained in the GA prediction from the pathological population are much larger than those obtained from the neurotypical population. An exhaustive visualization of the results obtained from each descriptor is reported in the Supplementary Figures S1, S2, which shows larger prediction errors and a similar trend for both neurotypical and pathological populations.

**Figure 5:**
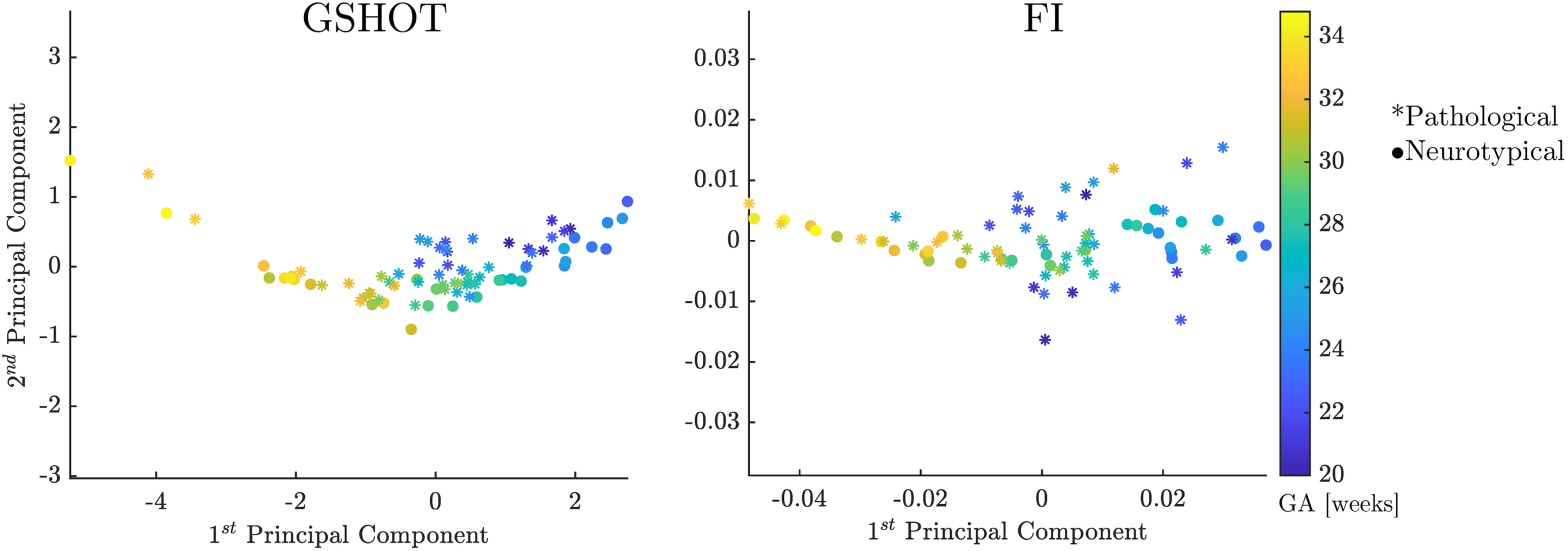
GSHOT and FI first and second principal components. Neurotypical fetuses are represented by points, while pathological (spina bifida) fetuses are represented by stars. The colorbar displays the color code used for the fetus GA ranges. These results are derived from Payette et al. (19) dataset.

PCA analysis shows that shape descriptors codify the largest part of the relevant information about the inner surface of the CP in the first few components. Among all the shape descriptors, GSHOT and FI provide the best performances. Their combined first two components explain 98.8% of the variability in the FeTA neurotypical fetuses and more than 95% in the FeTA pathological fetuses (Supplementary Table S1). Figure 5 shows that GSHOT provides a clear graphical representation of the fetal evolution. The different GA samples are distributed in a distinct “U” shape, with an increase from right to left on the first component (*x*-axis). The GA ranges behave symmetrically on the second component (*y*-axis), growing downwards on the negative axis values and upwards on the positive axis values. This geometric behavior interpretation cannot be inferred from FI results as it revealed lower discretization ability.

The other descriptors have a graphical representation worse than FI (see Supplementary Figure S3).

## 4 Discussion

Gyrification in the human fetus occurs from the 10th gestational week and continues hierarchically almost up until the last weeks of pregnancy (29). During gyrification, the smooth surface of the fetal brain develops folds and wrinkles, increasing the surface area of the cerebral cortex. This folding is essential for accommodating the large number of cortical neurons and connections within the limited space of the skull. However, disruptions or abnormalities during this process can lead to cortical malformations such as lissencephaly [smooth brain, (30)], or polymicrogyria [excessive folding, (31)]. These malformations are associated with various neurological disorders and cognitive impairments. Therefore, understanding its construction mechanism is crucial for studying brain development, function, and disorders (32).

Here, we introduced novel multidimensional point-wise shape signatures (HKS, WKS, SHOT) to analyze the inner cortical surface development in the fetal brain by an innovative procedure. These signatures are a well-established method in computer graphics to analyze the geometry of an object based on its spectral properties. However, they have rarely been applied in the medical field due to the intricate nature of medical data, the heavy workload and professional expertise required, and the need for thorough validation and approval processes to comply with regulatory standards (3, 33). We compared the shape properties of the brain's cortical structure extracted from multidimensional point-wise shape signatures with those obtained from scalar point-wise curvature-based signatures (C, H, K, SI, FI). The results obtained from the GA prediction through shape properties indicate that GSHOT is the most reliable shape descriptor. GSHOT accurately captures the morphological changes during fetal neurodevelopment, with an estimated error of less than a week, outperforming all other descriptors investigated. Figure 4 shows that this error remains consistent during gestation. Furthermore, Figure 5 highlights that the first principal component explains a large portion (93.2%) of all variability, proving its capability to encode changes in morphological shape. Consequently, this principal component alone can be considered the best representative summary of the fetal gyrification process. The GA prediction in pathological subjects (i.e., fetuses with spina bifida) tends to be overestimated, particularly in the earlier gestational weeks where the fetuses have not yet undergone spinal lesion repair surgery. Therefore, the lack of cerebrospinal fluid circulation can lead to abnormal pressure dynamics in the brain. This, in turn, may result in more pronounced or abnormal cortical folding. The difference between the predicted GA and the actual GA can serve as an indicator of pathology, making it easier to understand compared to the output of geometric analysis. This approach is similar to clinical practices in ultrasound, where each biometric measurement of the fetal brain is compared to the physiological range associated with the specified GA to identify any discrepancies. We can state that the implemented linear-SVR method is reliable, independently of fetal brain reconstruction method used (NiftyMIC, MIALSRTK, or Simple IRTK) thanks to the inclusion of all reconstruction typologies in the dataset used for training.

This study presents some limitations. First, other measures related to cortical folding, such as the gyrification index (34) and sulcal depth, can be studied. Furthermore, given cortical folding alterations associated with several pathologies (e.g., ventriculomegaly), cortical thickness is another measure worth investigating, considering partial volume effects. Second, other regression models (e.g., relevance vector regression RVR and Gaussian process regression GPR) can be tested by applying different kernel functions (e.g., polynomial, radial basis) to achieve the highest prediction performances. Third, we identified a larger prediction error in the pathological subset of the FeTA dataset used as test set. Unfortunately, we were not able to establish if this was caused by the clinical condition or by a model error. On the other hand, spina bifida is characterized by several malformative aspects both in the neonatal and in fetal central nervous system, encompassing not only altered gyrifications, but also altered brain and infratentorial structures size, and corpus callosum hypoplasia or partial dysgenesis (35–37). It is therefore reasonable to assume that the implemented shape descriptors highlighted a different developmental pattern in the gyrification process, leading to an error in the estimated GA that can be used as an indicator of pathology. Future works will investigate the brain's structure surface development across its different regions by using GSHOT to uncover new insight into the neurodevelopment process. Moreover, the quality of the T2w brain reconstructions and relative tissue label maps were not investigated as it is out of the scope of this study and has been previously addressed (19–21).

## 5 Conclusion

In this work, global multidimensional point-wise shape signatures (GHKS, GWKS, and GSHOT) are exploited to improve the prediction of GA in neurotypical and pathological fetuses.

GSHOT outperforms other global multidimensional point-wise signatures and scalar point-wise curvature-based signatures (C, H, K, SI, FI), providing researchers with a more sophisticated tool to capture more nuanced aspects of shapes. This approach enhances the accuracy and effectiveness of shape analysis tasks such as classification, segmentation, or matching, potentially leading to new methods for early detection of fetal diseases. In addition, a novel exploration of the fetal brain based on this approach can potentially uncover new insight into the structures development of the brain.

Finally, this innovative procedure for extracting multidimensional global descriptors from a given point-wise signature can also be applied in different scenarios of shape analysis within computer graphics.

## Data Availability

Publicly available datasets were analyzed in this study. This data can be found here: http://crl.med.harvard.edu/research/fetal_brain_atlas; https://gin.g-node.org/kcl_cdb/fetal_brain_mri_atlas; https://www.synapse.org/#!Synapse:syn25649159/wiki/610007.

## Footnotes

^1^ https://github.com/gift-surg/NiftyMIC

^2^ https://github.com/Medical-Image-Analysis-Laboratory/mialsuperresolutiontoolkit

^3^ https://gitlab.com/mariadeprez/irtk-simple
