# Supplementary material for: Fetal gestational age prediction via shape descriptors of cortical development
Source: Front Pediatr. 2024 Nov 20;12:1471080. doi: 10.3389/fped.2024.1471080 (PMC11614626; doi:10.3389/fped.2024.1471080)
Supplement: Supplementary file 1 [file Datasheet1.docx]

**SUPPLEMENTARY MATERIAL**


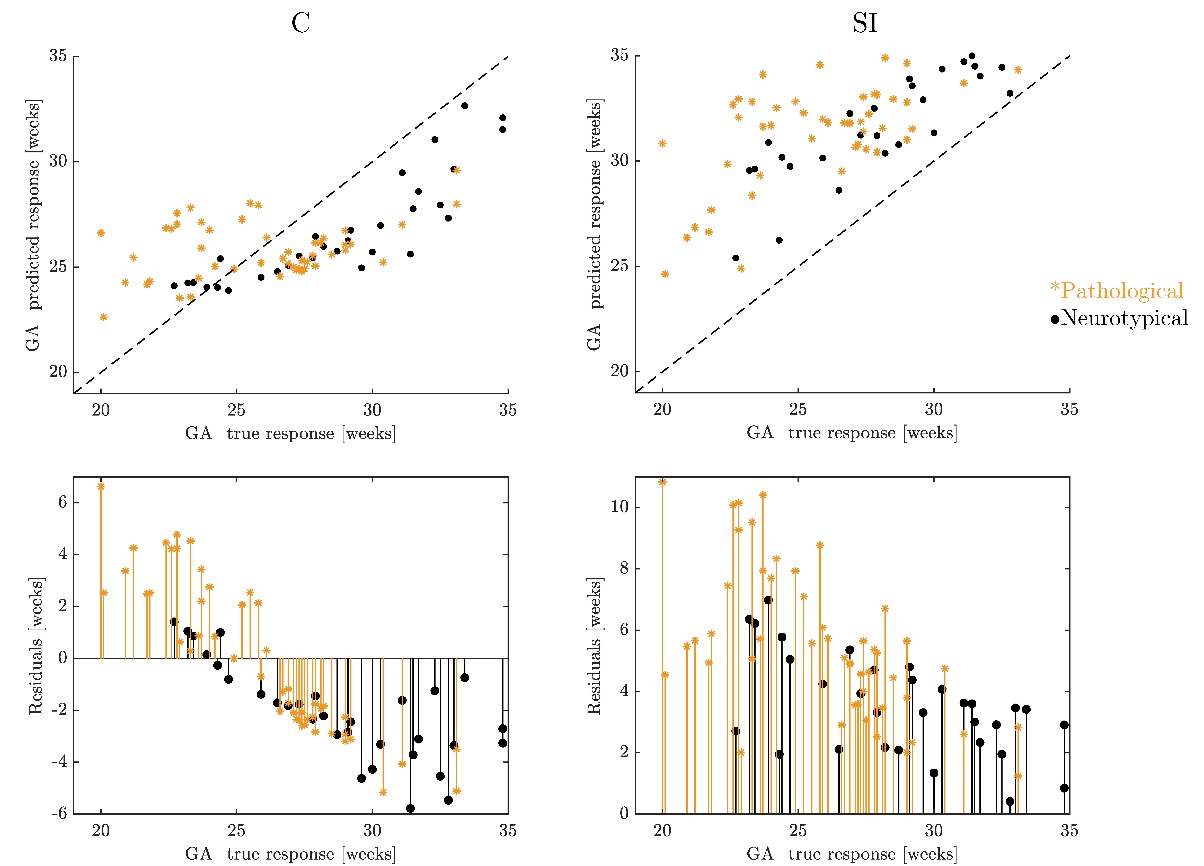


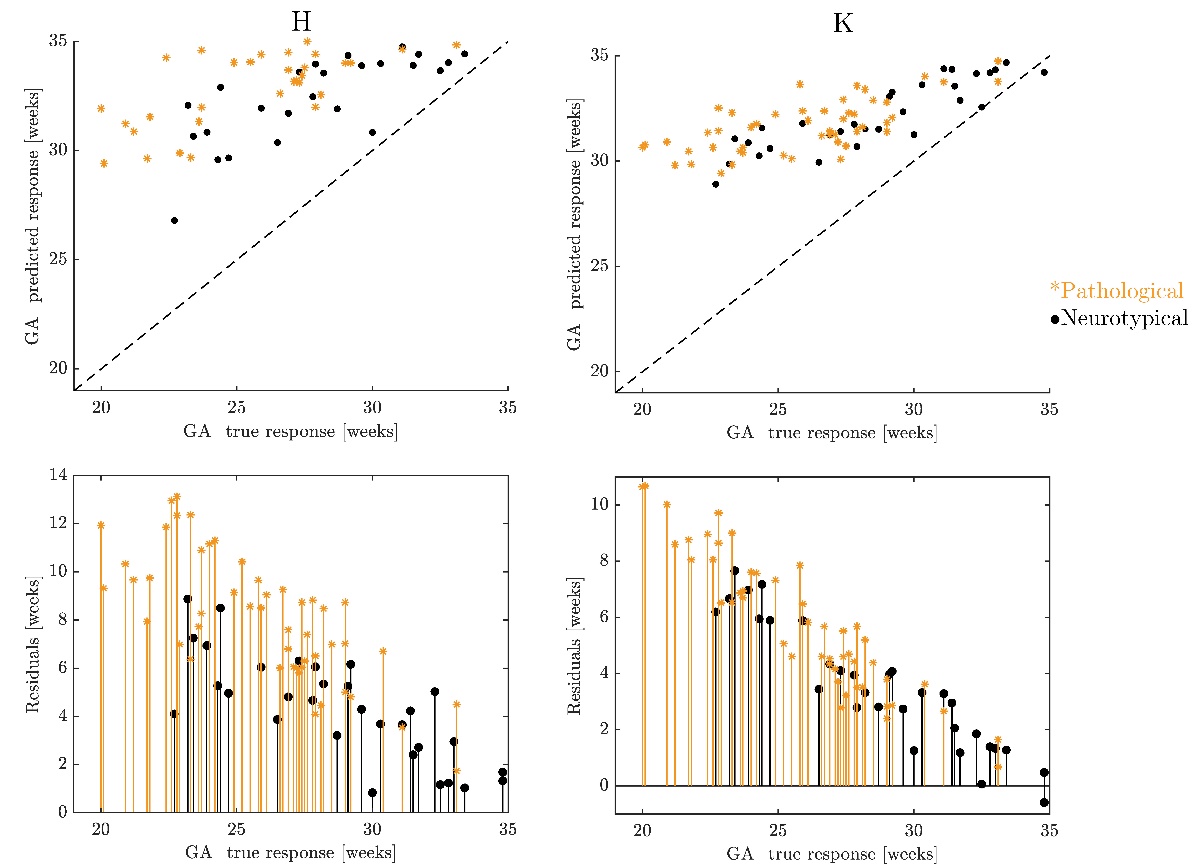


Supplementary Figure 1: A visualization of the results in GA prediction using scalar point-wise curvature-based signatures (C, SI, H, and K). The figure displays the true versus predicted response plots at the top, while the residual plots at the bottom. Black points represent neurotypical fetuses, while pathological (spina bifida) fetuses are described by blue stars. The FeTA dataset was adopted for the GA prediction (Payette et al., 2023).


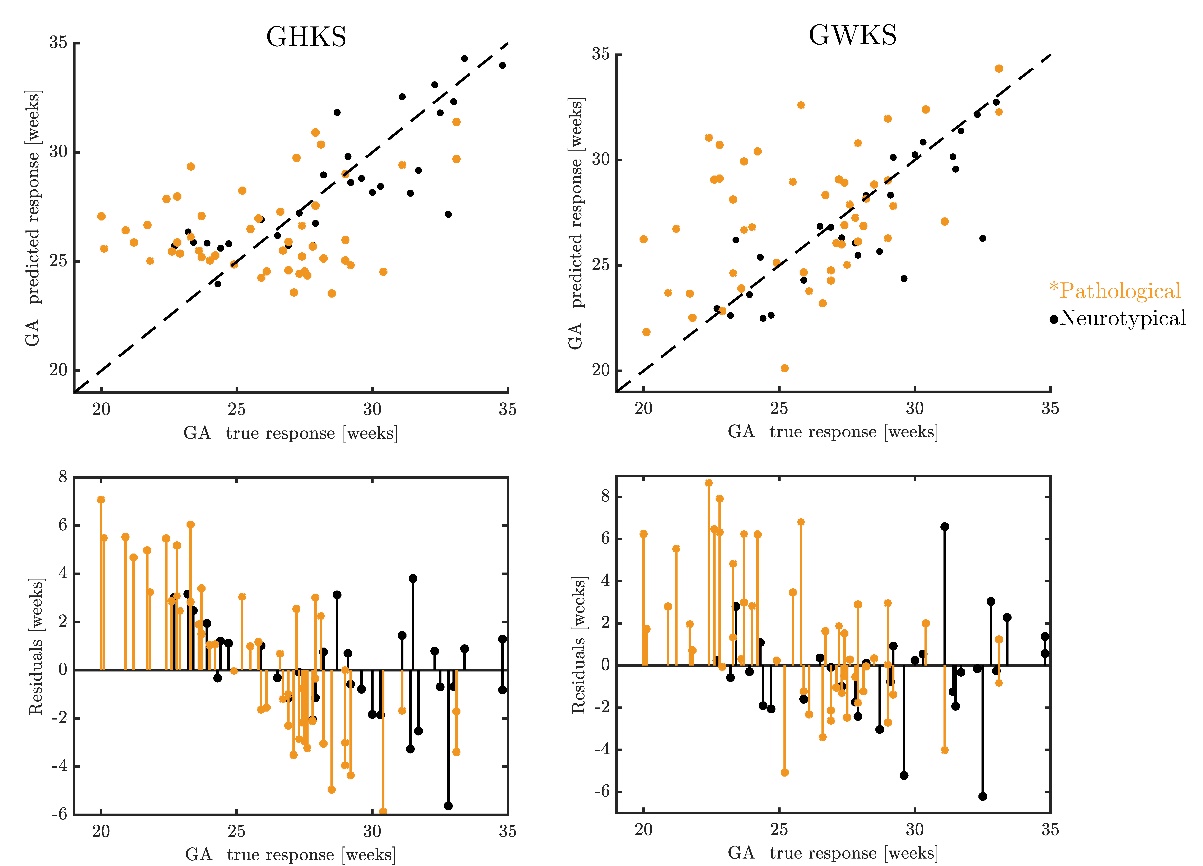


Supplementary Figure 2: A visualization of the results in GA prediction using global multidimensional point-wise shape signatures (HKS, and WKS). The figure displays the true versus predicted response plots at the top, while the residual plots at the bottom. Black points represent neurotypical fetuses, while pathological (spina bifida) fetuses are described by blue stars. The FeTA dataset was adopted for the GA prediction (Payette et al., 2023).


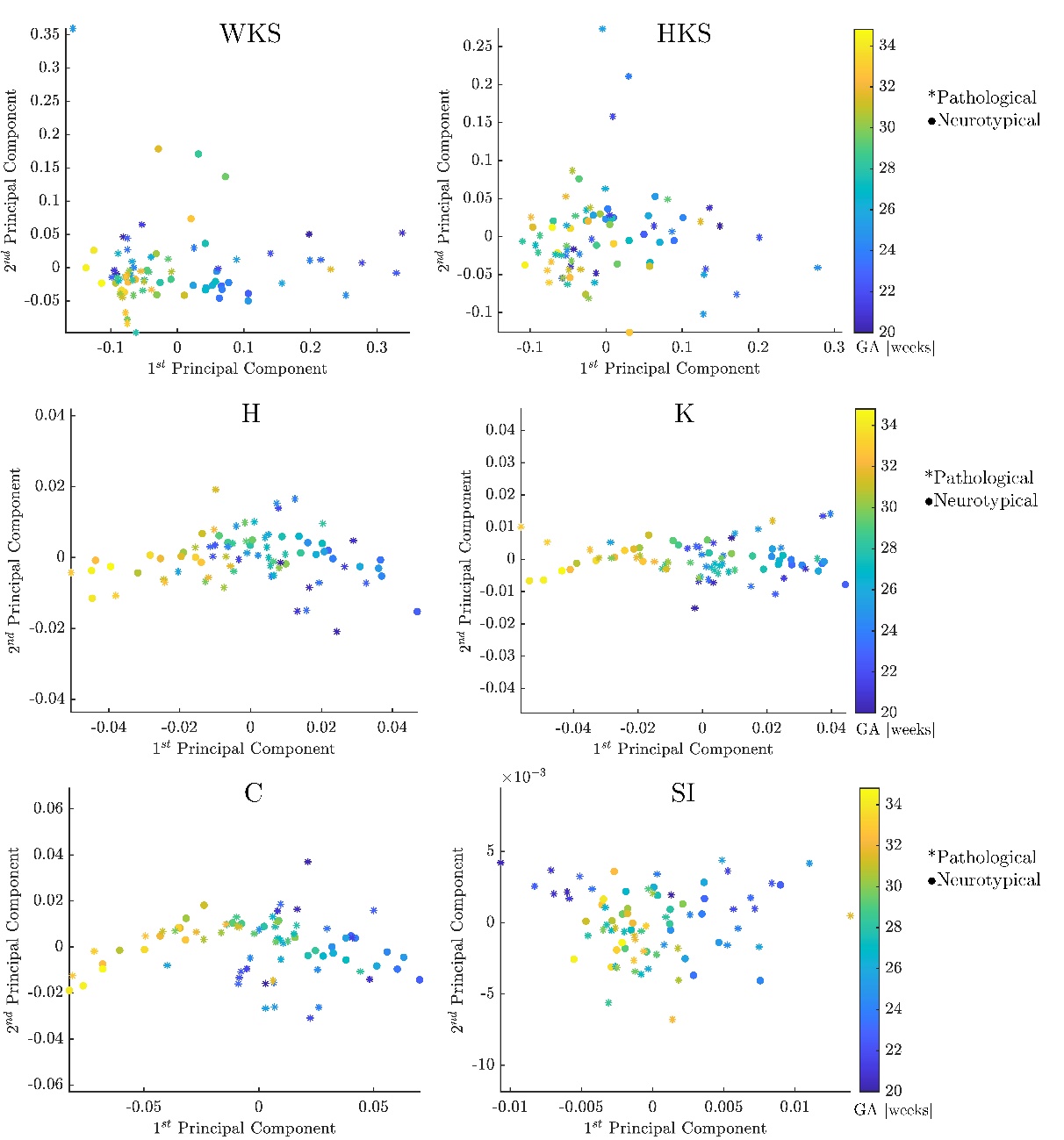


Supplementary Figure 3: Global multidimensional point-wise shape signatures (HKS, and WKS) and scalar point-wise curvature-based signatures (C, SI, H, and K) first and second principal components visualization. Neurotypical fetuses are represented by points, while pathological (spina bifida) fetuses are represented by stars. The colorbar displays the color code adopted to represent the fetuses' GAs. These results are derived from the quoted Payette et al. (2023) dataset.

Supplementary Table 1: Variability explained by the first two components of the Principal Component Analysis (PCA) conducted for GSHOT and FI. The results are shown for neurotypical and pathological fetuses.

| **PCA** | **GSHOT** | | **FI** | |
| --- | --- | --- | --- | --- |
|  | **Neurotypical fetuses** | **Pathological fetuses** | **Neurotypical fetuses** | **Pathological fetuses** |
| 1^st^ Principal Component | 93.2% | 86.2% | 97.8% | 83.9% |
| 2^nd^ Principal Component | 5.6% | 8.8% | 1% | 11.9% |
| Sum | 98.8% | 95.5% | 98.8% | 95.8% |
